# Efficacy and safety of artesunate plus amodiaquine in routine use for the treatment of uncomplicated malaria in Casamance, southern Sénégal

**DOI:** 10.1186/1475-2875-6-150

**Published:** 2007-11-15

**Authors:** Philippe Brasseur, Patrice Agnamey, Oumar Gaye, Michel Vaillant, Walter RJ Taylor, Piero L Olliaro

**Affiliations:** 1 UR 077, IRD, Dakar, Sénégal; 2 Laboratoire de Parasitologie-Mycologie CHU Amiens, France; 3 Faculté de Médecine, Université Cheikh Anta Diop, Dakar, Sénégal; 4 Clinical Epidemiology and Public Health Unit, Center for Health Studies, CRP-Santé, Luxembourg; 5 Unité 3677, Bases thérapeutiques des inflammations et infections, Université Victor Segalen Bordeaux 2, Bordeaux, France; 6 Travel and Migration Medicine Unit, Geneva University Hospital, Geneva, Switzerland; 7 UNICEF/UNDP/WB/WHO Special Programme for Research & Training in Tropical Diseases (TDR), 20 avenue Appia, CH1211 Geneva 27, Switzerland

## Abstract

**Background:**

There are no data on the long term use of an artemisinin combination treatment in moderate or high transmission areas of Africa.

**Methods and findings:**

Artesunate plus amodiaquine (AS+AQ) was used to treat slide-proven *Plasmodium falciparum*-infected patients of all ages in the Oussouye district, Casamance, Senegal, over a period of six years (2000 to 2005). Efficacy, by Kaplan Meier survival analysis (n = 966), and safety (adverse event rates, n = 752) were determined over 28 days. A weight-based dosing regimen was used for the loose tablets during 2000–2003 (n = 731) and a commercially available co-blister was used during 2004–2005 (n = 235).

Annual crude (non PCR corrected) rates remained stable over the study period [range 88.5–96.7%; overall 94.6 (95% CI 92.9–95.9)]. Nine co-blister treated patients (0.9%) withdrew because of drug-related adverse events; seven had gastrointestinal complaints of whom two were hospitalized for vomiting. By Day 28, the mean total bilirubin (n = 72), AST (n = 94) and ALT (n = 95) values decreased. Three patients had Day 28 AST/ALT values > 40 < 200 IU/L. Changes in white cell counts were unremarkable (n = 87).

**Conclusion:**

AS+AQ in combination was highly efficacious and well-tolerated in this area and justifies the decision to use it as first line treatment. Long-term monitoring of safety and efficacy should continue.

## Background

Artemisinin-containing combination therapies (ACTs) are now being deployed in some 42 malaria endemic countries and a further 26 have agreed to adopt ACTs following the World Health Organization (WHO) recommendation that ACTs should be the first line drugs for treating uncomplicated falciparum malaria (data provided by WHO/GMP, February 2007).

Artesunate plus amodiaquine (AS+AQ) is one of the currently available ACTs and is in use in Indonesia and 18 African countries (Burundi, Cameroon, Congo, Côte d'Ivoire, Democratic Republic of Congo, Equatorial Guinea, Gabon, Ghana, Guinea, Liberia, Madagascar, Malawi, Mauritania, Senegal, Sao Tome & Principe, Sierra Leone, Sudan (South), Zanzibar).

Because the WHO policy change is recent, there is, to date, little experience with the systematic use of these drugs in malaria endemic countries. The most reliable data regarding systematic use comes from the low transmission areas of the Thai Burmese border where artesunate plus mefloquine has been in continuous use since the early 1990s, well before the WHO recommendation. This combination has consistently produced high cure rates, achieved a reduction in the transmission of *Plasmodium falciparum *while the trend of increasing *in vitro *mefloquine resistance has been reversed [1,2]. Similar results have been reported with the deployment of artemether/lumefantrine (Coartem^®^) in another low-transmission area on the South Africa-Mozambique border, where the malaria burden has fallen and where there has been a reduction in morbidity and mortality [3].

By contrast, there are no reported data on the long term use of ACTs from areas of higher malaria transmission. Having this information is important because areas of moderate and high transmission account for most of the global malaria burden and experience from areas of low transmission may not necessarily be applied to higher transmission settings [4]. In addition, the useful therapeutic life spans of the ACTs may vary with transmission intensity and with such factors like cost, compliance and treatment seeking behaviour.

This paper reports on the safety and efficacy of AS+AQ in the chloroquine-resistant, Oussouye district of southern Casamance, Senegal, during 2000–2005 for treating patients with parasitologically confirmed falciparum malaria.

## Methods

### Study site characteristics

This study was conducted at the outpatient clinics of four dispensaries (Mlomp, Oussouye, Kabrousse and Djembereng) all situated in the District of Oussouye, southern Casamance. The total population of the district is circa 70,000, mostly farmers. Malaria transmission is perennial and meso-endemic with an increase in cases during the rainy season (July to December). The entomological inoculation rate is 25 infected bites per person-year [5].

In this area, the rate of chloroquine-resistant strains has remained stable at around 66% between 1997 and 2004 [6]. Despite this high rate, trials over the past decade have shown that AQ alone or combined with AS are efficacious [7-9].

### Study methodology

This was a non-comparative assessment of the efficacy and safety of AS plus AQ conducted over 28 days. Potentially eligible patients of all ages who attended the clinic with either a history of fever or a confirmed fever (measured axillary temperature ≥37.5°C) and a positive Giemsa-stained thick film for *P. falciparum *were briefed about the study and those who gave their written informed consent were registered and their houses were mapped. Entry criteria included: weight >5 kg; male and non-pregnant or breast-feeding female; living in the study area (for ease of follow-up); having given informed consent to participate; fever or history of fever; falciparum parasitaemia 1,000–200,000 parasites/μL; no antimalarial drug intake in the previous week; able to take oral drugs; no signs/symptoms of severe malaria; no major intercurrent illness or history of cardiac, hepatic or renal disorder; no known allergy to study drugs. The study was approved by the Senegalese National Ethical Committee.

All eligible patients were treated with AS + AQ. Initially (2000–2001), the use of AS+AQ was restricted to the rainy season, and later extended (2002–2005) to all year round. During 2000–2003, loose tablets were used in combination and patients were dosed by body weight. Subsequently a blister pack containing both drugs became available during 2004–2005 and patients were dosed by age (except 30 subjects in 2004 who were dosed by weight). The drugs used were:

▪ loose combination: Arsumax^® ^tablets (Sanofi-Aventis) containing 50 mg of AS and Camoquin^® ^tablets (Parke-Davis) containing 200 mg of AQ base tablets. The doses used were 4 mg/kg/day (AS) and 10 mg/kg/day (AQ) both for three days.

▪ the blister pack: Arsumax^® ^tablets (Sanofi-Aventis) containing 50 mg of AS and AQ tablets (Sanofi-Aventis) containing 153 mg of AQ base. There were three dosing blisters and both drugs were administered following the manufacturer's instructions: (i) children below one year of age = 1/2 tablet of each drug; (ii) children between one and six years of age = one tablet of each drug; (iii) children above six and below 13 years of age = two tablets of each drug, and (iv) above 13 years of age = four tablets of each drug.

All treatments were administered under supervision in the clinic, except in 2005 when only the first dose was supervised and patients were instructed to continue their treatment at home. Patients were seen on Days 0–3 inclusive during 2000–04 (Days 0, 2 and 3 in 2005), and then on Days 7, 14, 21, 28 or in between, as needed. Non attendees for scheduled clinic visits were actively sought by community health workers. Giemsa-stained thick films were read by trained microscopists and confirmed by one of the investigators (PB).

### Study end points

The end point for efficacy was the Day 28 crude cure rate calculated using Kaplan-Meier survival analysis [10] on the Intent-to-Treat (ITT) dataset (all patients who entered the study) for the whole period under study and by year; the log rank test was used to test for significance between years. Success was defined as parasite clearance that was sustained through Day 28. Failure to clear parasites and recurrent parasitaemia were considered as failures. Genotyping parasites was not done to distinguish between recrudescences and reinfections. All study withdrawals due to adverse events, irrespective of their relationship to study drug, were considered as failures. All patients lost to follow up were censored on the date they were last seen. Parasitological failures were rescued with quinine.

Safety was assessed by: (i) recording treatment emergent sign/symptom (TESS, i.e. events which were not present pre-treatment or worsened with treatment) and (ii) measuring liver (alanine, ALT and aspartic, AST transaminases and bilirubin), renal functions (creatinine) (KONELAB 60I analyser, Konelab, Finland) and haematology (haematocrit, WBC total counts), aiming for about 30% of the patients enrolled to have at least one baseline and one post-treatment sample. The common toxicity criteria for adverse events (CTCAE Version 3.0 08/09/2006) were used to evaluate and grade the severity of clinical events and laboratory measurements. Shift tables were done to show changes in severity of CTC grades between Day 0 and Day 28.

### Data analyses

Data were recorded in a case record form comprising demography, parasite counts, signs and symptoms, laboratory data and adverse events at each scheduled visit. They were double keyed in Excel^® ^using an end-user formatted sheet with online edit-checks.

Descriptive statistics are presented as counts, percentages, means and standard deviations, as appropriate. One-way ANOVA was used to assess the comparability of the patients' baseline characteristics between years and sites. Continuous data were assessed for normality using the Kolmogorov-Smirnov test; if significant, data were log-transformed and analysed using the 't' test, if normally distributed. Otherwise the Mann-Witney U test was used for paired comparisons. Homogeneity of variance was assessed with the Bartlett test. The Welch adjusted ANOVA was carried out if variances were unequal. Between groups comparisons of continuous data were further investigated with the Tuckey-Kramer post hoc test using means (normally distributed data) or ranks (skewed data). Dichotomous variables were analysed using chi-squared or Fisher's exact tests (Freeman-Halton if more than two categories) test if the expected counts were lower than five in any cell.

Kaplan-Meier survival analysis was performed to evaluate cure rates: (i) for all the years combined, (ii) between years, and (iii) between the loose and the co-blistered products [10]. The log rank test was used to test for homogeneity between survival curves of each year and the loose and the co-blistered products. A Cox proportional hazard model of the probability of failure was done to test for the contribution of the year of treatment and products. A descending stepwise manual modelling strategy based on the likelihood ratio test between subsequent models was carried out, beginning with a saturated model containing all factors.

A p value of < 0.05 was considered statistically significant. All tests were two-tailed. Statistical analyses were conducted with the statistical package SAS version 9.3.1 (SAS Institute, Cary, NC, USA)

Because the dosing of AS and AQ changed during the study, the doses of AS and AQ taken by patients with the loose and the blister combinations are presented in relation to two dosing schedules: (i) the recommended weight based dosing of 4 mg/kg/d × 3 d for AS and 10 mg/kg/d × 3 d for AQ base, and (ii) the new age based dosing regimen with newly defined therapeutic windows of 2–10 and 7.5–15 mg/kg/d, respectively [11].

## Results

### Baseline characteristics

During 2000–2005, 966 patients were enrolled, of whom 723 in Mlomp (75%), 110 in Diembereng, 27 in Kabrousse and 106 in Oussouye. The loose combination was given to 731 patients during 2000–03 and the co-blistered product to 235 patients during 2004–05. The dose was calculated on body weight for 761 patients (731 treated with the loose and 30 with the co-blistered products) and on age for 205 (all co-blister). Treatment was given supervised to 810 patients and unsupervised to 156 (co-blistered product in 2005). Table 1 shows the patients' baseline characteristics. Overall, for all the years combined, there were 30% more male than female patients, the mean age, weight and temperature values were 13.8 years, 33.3 kg and 38.4°C. The geometric mean baseline parasitaemia was 31,850/μL. The mean (± SD) daily doses of AQ and AS for all the study years were 359 (± 179) mg and 131 (± 66) mg, respectively.

**Table 1:** Patient's baseline characteristics overall and by year of enrolment

	**year**	**2000**	**2001**	**2002**	**2003**	**2004**	**2005**	**2000–05**
Enrolled	N	214	302	117	98	79	156	966
Sex ratio	F/M	0.6	0.92	0.75	0.69	1.03	0.7	0.77
Age (years)	mean	12.8	12.9	14	16.8	15.7	14	13.8
	std	9.5	10.5	11.6	14.8	9.8	11.9	11.2
Body weight (kg)	mean	31.9	31	33.3	38	38.9	33.8	33.3
	std	15.8	16.3	18	20.4	16.6	17.8	17.3
Parasites/μL	geomean	32866	48098	24775	24394	39679	17408	31850
	std	3.7	3.6	4.8	4.7	3.4	4.7	4.2
Body temp. (°C)	mean	38.1	38.7	38.6	38.6	37.8	38.3	38.4
	std	1.1	0.6	0.9	1	1.2	1.2	1
AQ dose (mg/d)	mean	333	320	351	414	437	399	359
	std	156	152	178	242	170	189	179
AS dose (mg/d)	mean	128	124	134	140	155	130	131
	std	64	65	71	73	59	62	66

There were significant statistical differences for certain baseline parameters between most of the years. In particular, there was a difference in (i) body weight between 2000–2004, 2001–2003, 2001–2004, (ii) AQ dose between 2000–2004, 2000–2005, 2001–2003, 2001–2004, 2001–2005, 2002–2004, 2002–2005, and (iii) AS dose between 2001–2004 and 2004–2005.

**Figure 1:**
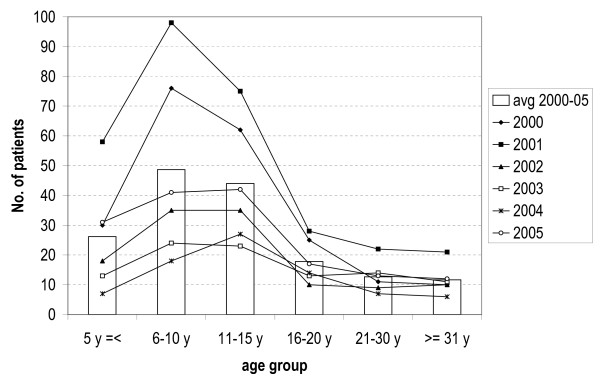
Age structure of the malaria cases treated by year of enrolment and mean over 2000–05.

The age structure of the population treated is presented in Figure 1. Overall, 16% of patients were ≤ 5 years of age, 30% were 6–10 years of age, 27% were 11–15 years of age, 11% were 16–20, 8% were 21–30 and 7% ≥ 30 years of age. Using these categories there was no difference between years and sites (p > 0.05). Just over half, 57% (n = 556), of the patients were aged between 6–15 years; 46% (n = 449) were under 11 years of age.

**Figure 2:**
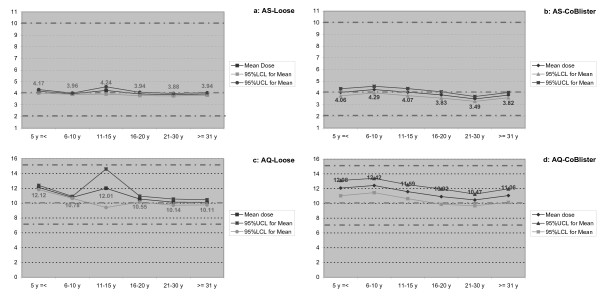
Mean (95% CI) doses of AS and AQ taken by patients treated with the weight based loose and aged based co-blistered drug regimens as a function of age. a = AS-Loose, b = AS-Blister, c = AQ-Loose, d = AQ-Blister.

The doses of AS and AQ taken by patients compared with the weight- and age-based dosing regimens are shown in Figure 2. With both products used, doses were well within the newly defined, therapeutic windows for both drugs. For AS, doses with the loose product and the co-blistered product were similar and very close to the target dose of 4 mg/day; the co-blister mean doses were slightly lower with wider 95% CIs. For AQ, doses were higher with both products than the target dose of 10 mg/day with a tighter 95% CIs for the loose combination except for 11–15 years old.

### Efficacy evaluation

**Figure 3:**
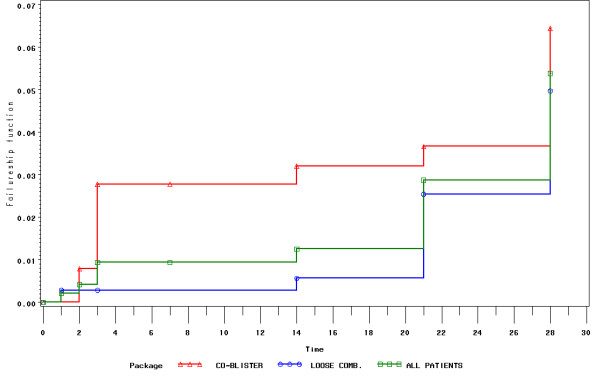
Kaplan-Meier of one minus survival curves to show cumulative parasitological failure rates overall (2000–05) and by year of treatment (all ages combined).

The Kaplan-Meier estimates of the crude cure efficacy rate was 94.6% (95% CI 93.0; 95.9) for all years combined. By individual years cure rates were 96.7% [93.2; 98.4] in 2000, 94.0% [90.6; 96.2] in 2001, 95.7% [90.0; 98.2] in 2002, 94.9% [88.2; 97.8] in 2003, 88.5% [79.0; 93.8] in 2004 and 95.9% [91.1; 98.1] in 2005. There were no differences (p = 0.12) in cure rates between years by the log rank test for homogeneity over time (Figure 3) All patients cleared their parasites by Day 3 (no early treatment failure, ETF); 36 patients returned with parasites during follow-up (late treatment failures, LTF) and nine were withdrawn due to an adverse event (considered as failures in our analysis – see below); 32 were lost to follow-up (censored) (Table 2). All treatment failures were retreated successfully with injectable quinine. All 36 LTFs occurred in patients under 16 years of age (20 in the age range 6–10). Efficacy was 95% in 0–10 years and 97% in 11 and above (log rank test, p = 0.03). In 2005 the losses to follow up amounted to 15%, while they were ≤3% in the other years.

**Table 2:** Distribution of treatment failures and losses to follow-up by year of study.

**(a) by year**	**2000**	**2001**	**2002**	**2003**	**2004**	**2005**	**2000–05**
**KM estimate of success (95%CI)**	**96.7 (93.2–98.4)**	**94 (90.6–96.2)**	**95.7 (90.0–98.2)**	**94.9 (88.2–97.8)**	**88.5 (79.0–93.8)**	**95.9 (91.1–98.1)**	**94.6 (93.0–95.9)**

LTF withdrawn due to AE lost to follow up	7	17	2	4	5	1	36
	0	0	0	0	4	5	9
	2	1	3	2	1	23	32

Treatment failures were late parasitological failures [LTF] and withdrawals in the Kaplan-Meier analysis. Losses to follow-up were censored in the KM analysis. See KM analysis in figure 3.

The Kaplan-Meier estimates of the crude cure efficacy rates were similar (p = 0.14) between the loose (n = 731) and the co-blistered (n = 235) products: 95.1% (93.3;96.5) vs. 93.1% (88.8;95.8), respectively. This held true whether dosing was weight (n = 761) or age (n = 205) based: 94.8% (92.9;96.2) vs. 94.2% (89.7;96.7), respectively (p = 0.51) and whether treatment was supervised (n = 810) or unsupervised (n = 156): 94.5% (92.7;95.9) vs. 95.9% (91.1;98.1), respectively (p = 0.75).

Year of study and product used were non significant contributors to failure in a Cox proportional hazard model. Using a saturated model containing year, product, site, age, sex, weight, dose AS and dose AQ, the contribution to hazard of failure was border-line for year (p = 0.06) but significant for the total daily dose of AS (p = 0.004) for a hazard of failure of 0.993 [0.988; 0.998]. Only year 2004 was statistically different from the reference year 2000 (p = 0.003): the hazard of failure was four times greater in 2004 than in 2000 (hazard ratio = 4.496 95% CI = [1.661; 12.168]).

### Safety evaluation

Complete safety records are available for 752 patients enrolled during 2001–2005. At presentation (Day 0), all patients reported fever or had a measured fever in the clinic. Other malaria associated symptoms/signs on presentation were weakness [n = 296 (29%)], headache [n = 291 (29%)], vomiting [n = 133 (13%)] and nausea [n = 113 (11%)]. There were no significant differences in the frequency of symptoms/signs between the different age groups or sexes.

After treatment, 69 patients (7.1%) experienced at least one treatment emergent sign/symptom (TESS) which was either not present pre-treatment or worsened post-treatment: 54 patients suffered one TESS, 14 had two and 1 had three for a total of 85 TESSs: 36 were vomiting, 19 vertigo, 11 asthenia, 8 pruritus without a rash, 5 abdominal pain, 3 diarrhoea, 2 headaches and 1 nausea (Table 3). TESSs were more likely to be reported by the co-blister recipients: 54 (5.6%) vs. 15 (1.5%) (p < 0.0001) and were independent of age. For the co-blister 39 patients suffered one TESS during the study, 14 suffered 2 TESSs and one suffered 3 TESSs; of patients treated with the loose combination, 15 suffered one TESSs (p < 0.0001).

**Table 3:** Type, frequency and severity of Treatment Emergent Signs and Symptoms (TESS) and number of patients experiencing at least one episode

**Symptom**	**Intensity**				**Total n(%) of TESS**
		
	**Mild**	**Moderate**	**Severe**	**Very severe**	
Abdominal pain		5 (10.2%)			5 (5.9%)
Asthenia		2 (4.1%)	9 (53%)		11(13%)
Diarrhoea		3 (6.1%)			3 (3.5%)
Headache		1 (2%)	1 (5.9%)		2 (2.4%)
Nausea				1 (33.3%)	1 (1.2%)
Pruritus		4 (8.2%)	3 (17.6%)	1 (33.3%)	8 (9.4%)
Vertigo	4 (25.%)	13 (26.5%)	2 (11.8%)		19 (22.3%)
Vomiting	12 (75%)	21 (42.9%)	2 (11.8%)	1 (33.3%)	36 (42.3%)

**Total**	16 (18.9%)	49 (57.6%)	17 (20%)	3 (3.5%)	85 (100%)

Nine patients, all treated with the co-blistered product (two weight-based and seven age-based), were withdrawn from the study because of a TESS for an overall, crude withdrawal rate of 0.9%: 3.8% [(co-blister) vs. 0% (loose), p < 0.0001(Table 4)]. All events were considered probably related to AS+AQ except one case of vomiting (possibly related). In five such cases, the daily dose of AQ exceeded the target dose by 20% or more. Two patients with Grade 2 vomiting were admitted to hospital for intravenous quinine and symptomatic treatment; they recovered well. These hospitalizations define these AEs as serious adverse events (SAEs).

**Table 4:** TESS requiring withdrawal from the study. All patients received the co-blistered product.

										Dose (mg/d)				
										
										AS		AQ		
pt#	dosed by	super vised	day withdrawn	reason	AE (Day = grade)	imputability	measure taken	Age (Years)	Weight (Kg)	Actual	Target	Actual	Target	Δ %

108	age	yes	3	vomiting	D0 = 2; D1 = 2; D2 = 1	possible	metopimazine i.m.	19	49	200	196	612	490	20%
167	age	yes	2	vomiting	D1 = 2	probable	metopimazine i.m. + quinine i.m.	18	47	200	188	612	470	23%
186	weight	yes	2	abdominal pain	D1 = 2	probable	phloroglucinol p.o+quinine i.m.	19	77	200	308	612	770	-26%
212	weight	yes	2	abdominal pain + weakness	D1 = 2	probable	phloroglucinol p.o+quinine i.m.	14	49	200	196	612	490	20%
266	age	no	3	vomiting	D1 = 2	probable	hospitalized: metopimazine i.v. + quinine i.v.	11	36	100	144	306	360	-18%
464	age	no	3	vomiting	D1 = 2	probable	hospitalized: metopimazine i.v. + quinine i.v.	1 1/2	8.8	50	35.2	153	88	42%
116	age	no	2	vomiting	D1 = 2	probable	metopimazine i.m. + quinine i.m.	18	47	200	188	612	470	23%
8	age	no	3	vertigo	D1 = 2, D2 = 2	probable	none	16	53	200	212	612	530	13%
87	age	no	3	pruritus	D1 = 2, D2 = 2, D3 = 2	probable	dexchlorphe - niramine p.o.	16	59	200	236	612	590	4%

D0 = pre-treatment

For the laboratory investigations, pretreatment results were available in 33%, 22% and 17% of patients for haematocrit (Hct), total white blood cells (WBC) and biochemistry, respectively (Table 5). No CTC grade 4 values were present at baseline and at Day 28. One patient had a grade 4 creatinine value on Day 7 [16.5 > 6 × ULN, ULN = 1,20 mg/dL] and returned to grade 0 at Day 28.

**Table 5:** Clinical laboratory values on D0 (pre-treatment), Day 7 and Day 28 (mean, standard deviation) and mean (95CI) changes between Day 0 and Day 28

					Difference vs. D0				
		N	mean	std dev	N	Mean	95% CI		Paired t-test

Haematocrit	D 0	323	39	6					
	D 7	233	36.3	5.6	228	-2.5	-3.2	-1.8	<.0001
	D 28	171	38.8	5	168	-0.3	-1.2	0.6	0.51
WBCs	D 0	212	6421	2929					
	D 7	120	6746	2509	120	-78.9	-602.6	444.8	0.76
	D 28	88	6987	2598	86	395.5	-234.8	1026	0.22
ASAT	D 0	167	40.2	38.8					
	D 7	134	23	41	123	-7.4	-14.4	-0.3	0.04
	D 28	106	21	22	101	-15.2	-22.1	-8.3	<.0001
ALAT	D 0	169	21.4	16.3					
	D 7	133	15	29.9	126	-4	-9.2	1.1	0.12
	D 28	105	11.7	11.7	103	-9.4	-12.7	-6.2	<.0001
Creatinine	D 0	166	0.6	0.3					
	D 7	132	0.8	1.4	118	0.2	-0.1	0.4	0.23
	D 28	106	0.7	0.3	100	0.1	0	0.2	0.02
Bilirubin	D 0	156	7	6.2					
	D 7	123	3.3	2.2	104	-3.9	-5	-2.8	<.0001
	D 28	75	4.2	4.3	71	-3.4	-5.1	-1.6	0.0004

D0 = pre-treatment.

std dev = Standard Deviation.

95% CI = Confidence Interval at 95% level for estimated mean change

There were no shifts between Day 0 and Day 28 from grades 0 – 2 to grades 3 or 4 (Table 6). Out of 12 changes in the total WBCs, three patients changed from grade 0 to grade 1 and one patient from grade 0 to grade 2, while the remaining eight patients changed to lower grades. There were 28 changes in CTC grade for AST; four had increased grades, from grade 0 to 1 (n = 3) and from grade 0 to grade 2 (n = 1). Out of 29, only two increased shifts were observed with ALT; one from grade 0 to grade 1 and one from grade 1 to grade 2. One shift from grade 0 to grade 1 was observed for the serum creatinine. For total bilirubin there were one change in CTC grades from 0 to 1 and two from 0 to 2. There were also nine decreases from grade 1 to grade 0 and four decreases from grade 2 to 0.

**Table 6:** Shift tables of CTC grades from Day 0 to Day 28 by CTC grading

	**WBC Day 28**			**ASAT Day 28**			**ALAT Day 28**			**Creatinine Day 28**		**Total Bilirubin Day 28**		
**Day 0**	**G.0**	**G. 1**	**G.2**	**G.0**	**G.1**	**G.2**	**G.0**	**G.1**	**G.2**	**G.0**	**G.1**	**G.0**	**G.1**	**G.2**

**Grade 0**	61	3	1	63	3	1	84	1	0	23	1	53	1	2
**Grade 1**	5	2	0	20	5	0	17	0	1	0	0	9	0	0
**Grade 2**	1	0	1	5	4	0	0	0	0	0	0	4	0	1
**Grade 3**	1	0	1	0	0	0								

CTC grading: G0 = none; G1 = mild; G2 = moderate; G3 = severe; G4 = very severe

**Figure 4:**
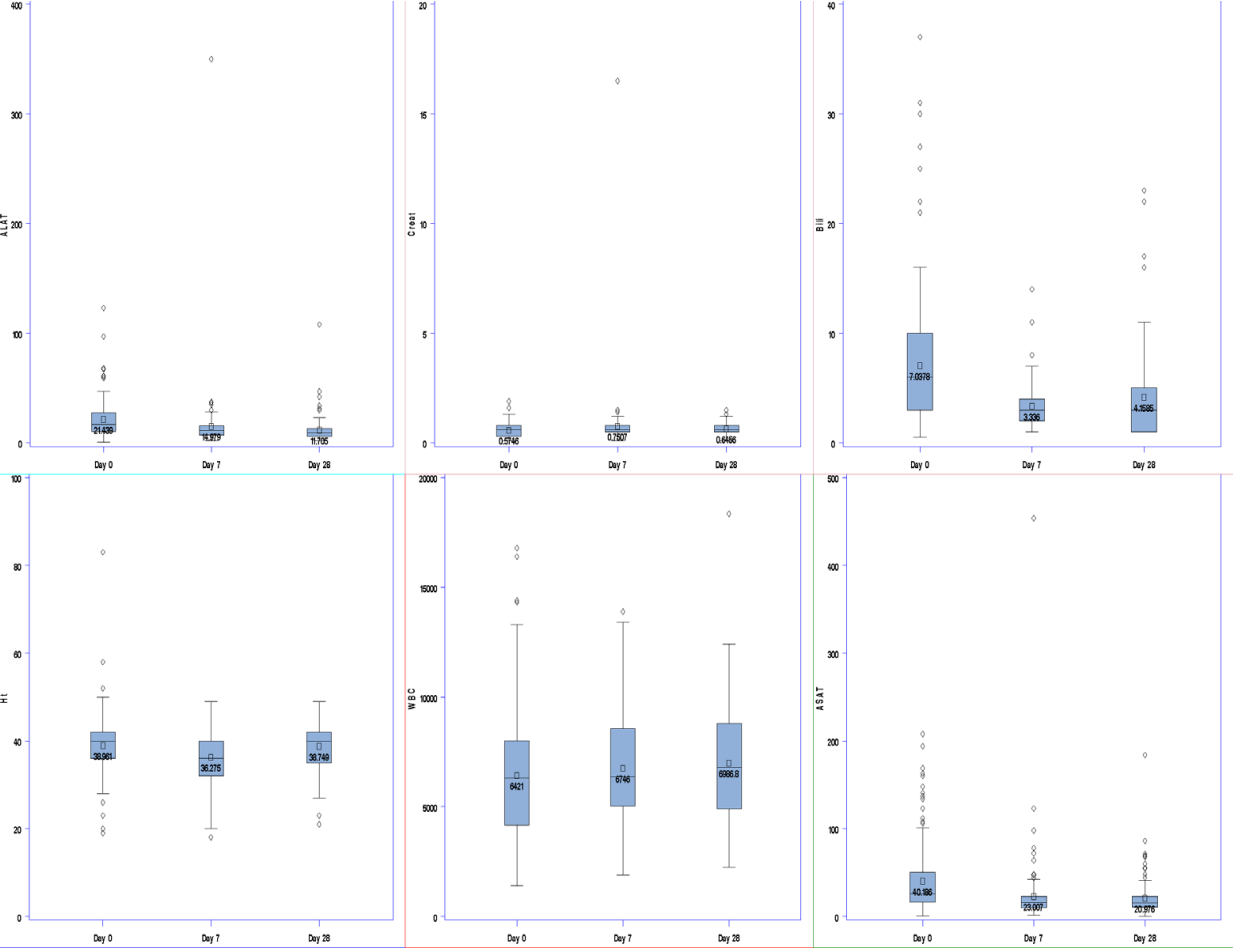
Boxplots of laboratory parameters over time. (ALT = aspartate transaminase; ALT = alanine transaminase).

There were no significant changes in mean values between Day 0 and Day 28 for the haematocrit (mean diff. 0.31 ± 6.10%, p = 0.51) and total WBC (mean diff. -395 ± 2940, p = 0.21) but there were significant decrease from Day 0 to Day 7 (2.52 ± 5.56%, p < 0.0001) and increase from Day 7 to Day 28 (-1.91 ± 4.70%, p < 0.0001) for haematocrit. The mean AST, ALT and bilirubin decreased significantly while there was a small (0.16 mg/dL) but statistically significant increase in the mean creatinine values between Day 0 and Day 28 (Figure 4).

## Discussion

The favourable results of this study support the policy decision of using AS+AQ in Senegal. It is premature to speculate how long this treatment will last but after six years of use, AS+AQ is still highly effective in the district of Oussouye where the rate of *in vitro *chloroquine resistance is > 60% [12]. The patients from this study represent approximately one third of the more than 3000 patients who have already received AS+AQ as part of its deployment as first-line treatment at the district level. Reassuringly, the *in vitro *susceptibility of *P*. *falciparum *parasites to desethyl-AQ and artemisinin has not changed under drug pressure during this initial phase of deployment [12].

The efficacy of AS+AQ was well above the 90% threshold recommended by the WHO despite the conservative approach used in analysing the data: ITT dataset, adverse events considered as treatment failures and no adjustment for PCR proven new infections. As such, this study reflects more the effectiveness of AS+AQ in the field, while probably underestimating the real efficacy of this treatment. Interestingly, failure rates even in children under 10 years of age were considerably lower than those detected in 1998 in the same age group [7]. It is difficult to explain these differences; in both cases, 28 day crude, non PCR-adjusted were measured, while patients were recruited during the rainy season in 1998 and all year round in 2000–05. Reinfections would be less frequent outside the rainy seasons; furthermore, the number of malaria cases has been decreasing after 2000.

Despite the good parasitological efficacy, treatment did not produce haematological recovery, a softer marker of efficacy. This may have been due to a combination of a relatively high mean, pretreatment haematocrit, the negative effect of AS on reticulocytes, and the short follow up period. Haematological recovery requires more than four weeks in some malaria settings [13]. Treatment was well-tolerated as testified by the very low withdrawal rate (<1%), including two hospital admissions, because of drug induced toxicity. No significant clinical laboratory toxicities were detected.

AS+AQ was equally effective whether given based on body weight as loose combination or by age as co-blistered products (as per the manufacturer's instructions); however, the latter induced more TESS and all the treatment withdrawals due to intolerance. The use of the co-blister did result in patients receiving doses of both drugs, in mg/kg, that were close to the recommended, weight based, mg/kg dosing regimen, though the co-blistered AQ mg/kg dose looked to be a little higher than the loose AQ and had a broader spread of the 95% CIs. Gastrointestinal complaints accounted for seven of the nine withdrawals and were probably AQ rather than AS related, given that AS has excellent tolerability. Five patients received AQ doses ≥20% than the weight based dose of 10 mg/kg and four were within the 15 mg/kg upper limit of the newly defined therapeutic window. Never the less, the AQ dose may have contributed to their gastro-intestinal complaints.

The results obtained when patients are dosed more loosely by age are important because more practical regimens may expose patients to drug doses that are outside of the recommended, strictly defined, weight based doses. A more refined practical dosing has been developed for a new, aged dosed, fixed dose combination of AS+AQ [14]. It is generally accepted that making treatments easier to understand and use by patients or e.g. their parents results in better compliance and that using fixed dose combinations enhances this [15,16]. The AS-AQ fixed-dose combination will become available later in 2007 and plans are afoot to test it in Casamance. Its tolerability will be evaluated.

No cases of hepatitis or severe leukopenia (as a surrogate marker of neutropaenia) were detected, the two amodiaquine-associated toxicities that have caused fatalities in the past, when used as prophylaxis in travelers [16]. However, the number of closely monitored patients was too low to detect rare toxicities, and differential WBC counts could not be done. Intensified monitoring of possible AS+AQ related AEs needs to be conducted in parallel with its widespread deployment, a practice which should be adopted systematically with all other ACTs.

This study confirms that, in this area of moderate/intense transmission, the risk of clinical malaria is present throughout life and that children between 6–15 years of age are most affected. This is important as clinical studies in these areas normally enroll patients up to 10 years old, and thus would miss an important segment of the patient population. In this study, although treatment was statistically less efficacious in children under 11 years of age than in older patients, efficacy rates were still high in both, 95% and 97%, respectively.

To conclude, this field study has shown a high and stable cure rate for AS+AQ even when dosed by age. Tolerability was good but continued intensive safety monitoring is still required in large numbers of patients for this ACT. Further research is planned to continue on the AS/AQ fixed dose combination.

## Competing interests

The author(s) declare that they have no competing interests.

## Authors' contributions

All authors read and approved the final manuscript.

• P Brasseur was the Principal Investigator of the study. He contributed to the concept, protocol, analysis and reporting of the study, and contributed to the preparation of the manuscript. He personally contributed to the treatment, follow-up of patients and quality control of the study.

• P Agnamey contributed personally to the treatment and follow-up of patients.

• O Gaye participated in designing the concept and protocol of the study and supervised study conduct.

• M Vaillant designed and conducted the analyses, contributed to the preparation of the manuscript.

WRJ Taylor and P Olliaro contributed to the concept of the project; design of the protocol and analyses, reporting of the study, and to the preparation of the manuscript.
